# Elastin Microfibril Interface-Located Protein 1 (EMILIN1) Mutation Mimicking Axonal Hereditary Motor Sensory Neuropathy

**DOI:** 10.7759/cureus.89357

**Published:** 2025-08-04

**Authors:** Somarajan Anandan, Sajeesh Rajendran, Ameena Sulaiman, Joesni Joy, Neethu Gopal

**Affiliations:** 1 Neurology, St. Joseph's Mission Hospital, Anchal, IND; 2 Neurology, Welcare Hospital, Ernakulam, IND; 3 Radiology, Mayo Clinic, Jacksonville, USA

**Keywords:** axonal hmsn, charcot marie tooth disease type 2, cmt, emilin 1, hereditary motor sensory neuropathy type 2, hmsn

## Abstract

Hereditary peripheral neuropathies may present as isolated neuropathy or as a part of a more complex neurological disorder. Hereditary motor sensory neuropathy is the most common form of hereditary neuropathy. The discovery of an increasing number of causative genes over the years has significantly complicated the classification of hereditary motor sensory neuropathy. Mutations in elastin microfibril interface-located protein 1 (EMILIN1) are linked to a range of connective tissue disorders, involving the vascular system, skeletal structures, and possibly the nervous system. We report a case of axonal hereditary motor sensory neuropathy associated with a mutation in the EMILIN1 gene.

## Introduction

Hereditary motor sensory neuropathy (HMSN), also known as Charcot-Marie-Tooth (CMT) disease [1], refers to a group of inherited peripheral nerve disorders characterized by progressive distally dominant motor and sensory impairment [2]. They commonly present with a characteristic phenotype of a length-dependent, isolated neuropathy progressing over decades [3]. Pathologically, they are characterized by demyelination, axonal degeneration, or a combination of both. Classification is based on clinical presentation, inheritance pattern, electrophysiological findings, metabolic abnormalities, and specific genetic markers. Most popular classifications are CMT1 (autosomal dominant, demyelinating), CMT2 (autosomal dominant or recessive, axonal), intermediate CMT (autosomal dominant or recessive), CMT4 (autosomal recessive, demyelinating), and CMTX (demyelinating or axonal, X-linked dominant or X-linked recessive) [4,5].

Elastin microfibril interface-located proteins (EMILINs), comprising EMILIN1, EMILIN2, and EMILIN3, are a family of extracellular glycoproteins that play a vital structural role within the elastin-fibrillin microfibril network and are key modulators of cellular signaling. They also contribute to tumor suppression and inflammatory regulation [6]. Mutations or dysfunctions in EMILINs have been associated with connective tissue disorders characterized by features such as increased skin elasticity, easy bruising without atrophic scarring, impaired coagulation, aortic aneurysms, joint hypermobility, and recurrent tendon ruptures [7]. Rarely, the EMILIN1 mutation can present as axonal HMSN with mild hypermobility of joints, as in the present case.

## Case presentation

**Figure 1:**
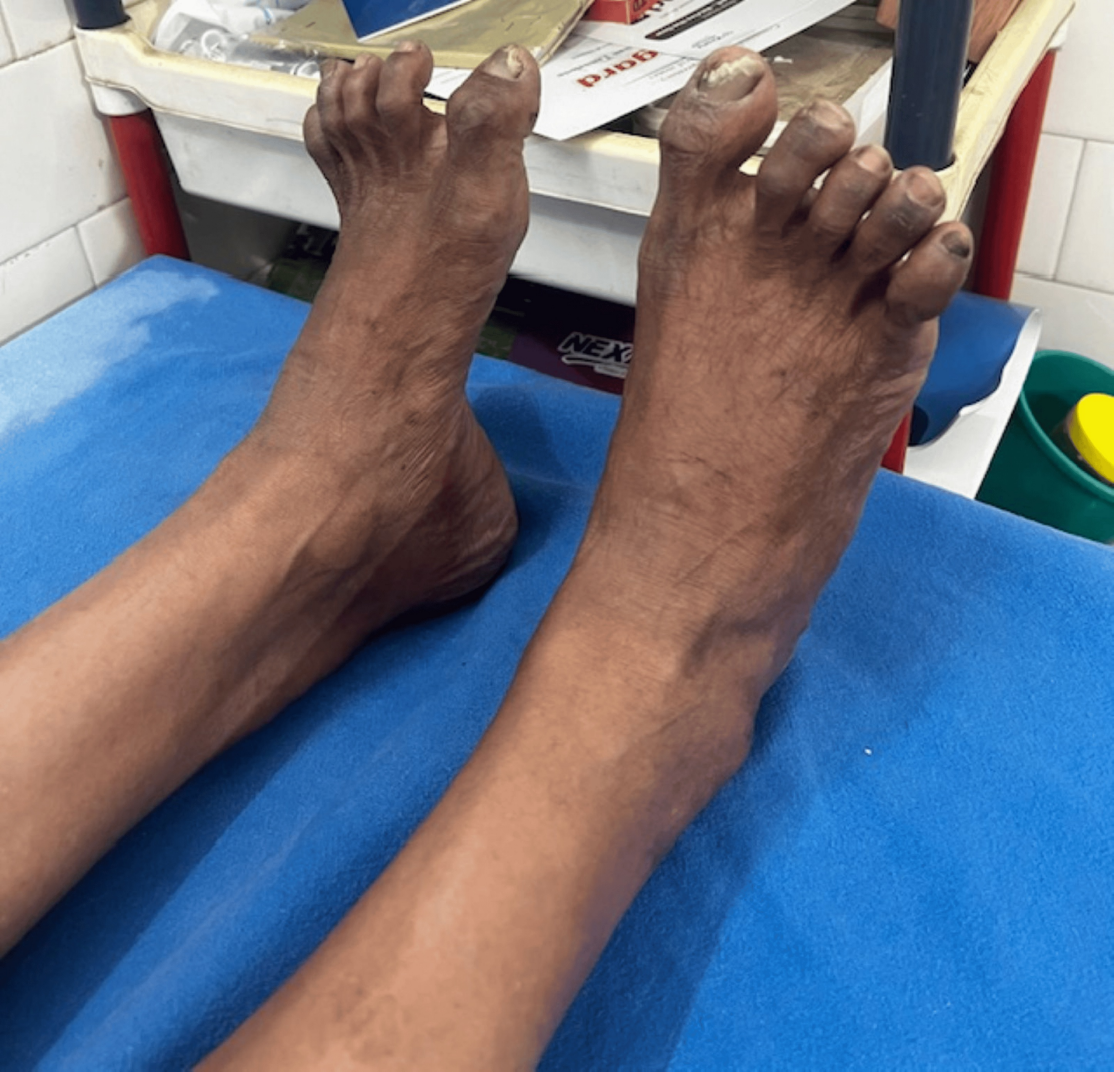
Pes cavus with hammer toes (proband).

We present a case of a 55-year-old female born of a non-consanguineous marriage. Her motor and speech developmental history was normal. She had delayed menarche and premature menopause at the age of 20 years. She has a history of recurrent bilateral patellar subluxation in her teens. Her neurological symptoms started at around 24 years of age, including slipping of footwear (chappals) from her feet without her knowledge, and difficulty in putting on footwear (chappals). She also noticed deformity of the feet at that age. There was no history of intellectual disability or cognitive decline. There were no cranial nerve symptoms. There was no upper limb weakness, wasting, or sensory symptoms. There was no history of any proximal muscle weakness. Her symptoms were insidiously progressive. She has a brother who has similar symptoms, which started at the age of 30 years. Her parents did not have any neurological symptoms or pes cavus. Her father died at the age of 70 years due to prostate cancer. General examination showed hypermobile joints. Neurological examination showed normal higher mental functions and cranial nerves. Upper limb examination did not show any weakness or wasting. Lower limb examination showed bilateral pes cavus (Figure 1).

There was foot dorsiflexion and plantar flexion weakness. Her extensor digitorum brevis was wasted. All her deep tendon reflexes were absent, and plantar reflexes were flexor. Sensory system examination showed absent vibration sense up to her knees. Joint position sense and pain sensations were reduced in the toes. Nerve conduction study was suggestive of axonal sensory-motor polyneuropathy (Tables 1, 2). Her brother was on follow-up from another hospital, and he has difficulty wearing footwear (chappals) and has foot deformity. His symptoms are also slowly progressive, and currently, he is ambulant without support. His records showed pes cavus, mild joint hypermobility, and generalized areflexia. His nerve conduction study was also suggestive of axonal sensory-motor polyneuropathy (Tables 3, 4). Clinical diagnosis was autosomal recessive CMT (AR-CMT), even though AR-CMT usually has a severe phenotype with onset in the first decade of life.

**Table 1:** Motor conduction study (proband). CMAP: compound muscle action potential

Nerve	Distal latency (ms)	CMAP amplitude (mV)	Conduction velocity (m/s)
Median right	3.23	8.4	51.17
Median left	3.44	12.1	54.92
Ulnar right	3.02	8	52
Ulnar left	2.81	8.5	55.44
Peroneal right	4.06	1	32.91
Peroneal left	In elicitable	-	-
Tibial right	In elicitable	-	-
Tibial left	In elicitable	-	-

**Table 2:** Sensory conduction study (proband). SNAP: sensory nerve action potential

Nerve	Peak latency (ms)	SNAP amplitude (µV)	Conduction velocity (m/s)
Median right	-	In elicitable	-
Median left	-	In elicitable	-
Ulnar right	2.54 (<3)	32	47.24
Ulnar left	2.88 (<3)	41.8	41.67
Sural right	-	In elicitable	-
Sural left	-	In elicitable	-
Superficial peroneal right	-	In elicitable	-
Superficial peroneal left	-	In elicitable	-

**Table 3:** Motor nerve conduction study (brother). CMAP: compound muscle action potential

Nerve	Distal latency (ms)	CMAP amplitude (mV)	Conduction velocity (m/s)
Median right	4.1	15.3	52
Median left	3.7	16.2	51
Ulnar right	2.9	13.2	52
Ulnar left	2.8	10.7	49
Peroneal right	5.4	0.5	37
Peroneal left	-	In elicitable	-
Tibial right	5.3	2.1	35
Tibial left	8.1	0.3	36

**Table 4:** Sensory nerve conduction study (brother). SNAP: sensory nerve action potential

Nerve	Peak latency (ms)	SNAP amplitude (µV)	Conduction velocity (m/s)
Median right	-	In elicitable	-
Median left	-	In elicitable	-
Ulnar right	-	In elicitable	-
Ulnar left	-	In elicitable	-
Sural right	-	In elicitable	-
Sural left	-	In elicitable	-
Superficial peroneal right	-	In elicitable	-
Superficial peroneal left	-	In elicitable	-

Her routine hematological and biochemical parameters were normal, including vitamin B12 levels. Blood was sent for next-generation sequencing, which revealed a heterozygous missense variant in exon 4 of the EMILIN1 gene (chr2:g.27083544A>G; depth: 141x) that results in the amino acid substitution of serine for asparagine at codon 658 (p.Asn658Ser; ENST00000380320.9). Her brother also tested positive for the same mutation at the same location (Table 5). Her mother, who has no neurological symptoms or signs, tested negative for the same mutation. After the genetic tests, she was diagnosed as autosomal dominant axonal CMT due to the EMILIN1 mutation. Her deceased father might have been affected by the same mutation, but with incomplete penetrance. There is no history of aortic aneurysm in her father or brother. After the genetic test results, she underwent echocardiography, which did not show any evidence of aortic aneurysm.

**Table 5:** Gene variant description. EMILIN1: elastin microfibril interface-located protein 1

Gene	Location	Variant	Zygosity	Disease	Inheritance	Classification
EMILIN1	Exon 4	c.1973A>G p.Asn658Ser	Heterozygous	Distal hereditary motor neuronopathy (HMN10) (OMIM 620080)	Autosomal dominant	Uncertain significance (PM2,PP3)

## Discussion

More than 100 genes have been identified as causative for Charcot-Marie-Tooth disease (CMT) and related disorders [8]. These genes encode proteins crucial for the structure of nerves, including components such as myelin, gap junctions, Schwann cells, and axons, as well as for nerve function, such as axonal transport and energy production. Inheritance patterns include autosomal dominant, autosomal recessive, X-linked recessive, and X-linked semi-dominant (with milder manifestations in carrier females) [1,5].

In demyelinating forms of hereditary motor and sensory neuropathy (HMSN), also known as CMT1 or CMT4, associated genes include GDAP1, MTMR2, SBF2, NDRG1, EGR2, SH3TC2, PRX, FGD4, PMP22, GJB1, MPZ, MFN2, MED25, and FIG4. For axonal forms (CMT2), implicated genes include LMNA, MED25, HINT1, GDAP1, LRSAM1, NEFL, HSPB1, MFN2, PLA2G6, PNKP, AIFM1, COA7, and KIF1A [2,4]. Among these, the most common mutations are PMP22 duplication, GJB1 mutations, PMP22 deletion, and MFN2 mutations.

Autosomal recessive forms of CMT (AR-CMT) generally present with earlier onset, typically before the age of two to three years, and progress rapidly, often leading to severe polyneuropathy. Affected individuals frequently exhibit pronounced distal limb deformities, such as pes equinovarus, claw-like hands, and significant spinal abnormalities. Genetic studies of AR-CMT have revealed multiple associated genes as follows: in demyelinating forms (AR-CMT1 or CMT4), these include GDAP1, MTMR2, SBF2, NDRG1, EGR2, SH3TC2, PRX, FGD4, and FIG4; and in axonal forms (AR-CMT2), the implicated genes are LMNA, MED25, HINT1, GDAP1, LRSAM1, NEFL, HSPB1, and MFN2 [9].

Elastin microfibril interface-located proteins (EMILINs) are extracellular matrix glycoproteins implicated in elastogenesis and cell proliferation [6]. Recently, a missense mutation in the EMILIN1 gene has been associated with an autosomal dominant connective tissue disorder and motor-sensory neuropathy in a single family [7,10]. There is evidence that autosomal dominant distal hereditary motor neuronopathy-10 (HMND10) is caused by a heterozygous mutation in the EMILIN1 gene on chromosome 2p23 [10]. There are reports of autosomal recessive EMILIN1 mutation presenting with short stature, tortuosity in multiple arteries, pulmonary stenosis, and multiple fractures [11].

There is a report of a family with distal motor neuropathy associated with the EMILIN1 mutation. Two patients were noted to have a low IQ, and two had minor gyration defects in the cingulate gyrus on brain imaging. None had evidence of a connective tissue disorder or involvement of other organ systems. These patients had normal SNAPs. Sural nerve biopsy displayed a reduction in myelinated fibers and non-specific changes in myelin morphology and thickness [10]. In some patients, it can be transmitted in an autosomal recessive pattern associated with global developmental delay in addition to polyneuropathy [12] or mimic cutis laxa type 1b [13].

Joint hypermobility is a well-described feature of the EMILIN1 mutation. EMILIN1 mutations could indirectly contribute to delayed puberty or menarche in some cases, due to their effect on blood vessel development in the reproductive organs. There are no reports of premature menopause in patients with the EMILIN1 mutations. As EMILIN proteins contribute to tumor suppression, it's possible that they may have an increased propensity to develop cancer, though it is not well characterized. Future case studies on EMILIN1 mutations should examine these aspects, and axonal HMSN with joint hypermobility may provide a clue to EMILIN1 mutations.

## Conclusions

Hereditary peripheral neuropathies may present as isolated neuropathy or as a part of a more complex neurological disorder. They can be axonal, demyelinating, or mixed in nature. Even though many genes are implicated in CMT, EMILIN1 mutation presenting as isolated axonal HMSN is extremely rare.

EMILIN1 gene mutations are usually associated with overt connective tissue disease, such as increased skin elasticity, joint hypermobility, and aortic aneurysm. Sensory motor polyneuropathy and hereditary distal motor neuronopathy can be rare manifestations of the EMILIN1 mutation. When a patient presents with HMSN phenotype and joint hypermobility, as in this case, an EMILIN1 mutation should be suspected, and next-generation sequencing may be considered. Future studies should investigate the development of aneurysms in these patients during follow-up.
